# Application of Silver Nanoparticles to Improve the Antibacterial Activity of Orthodontic Adhesives: An In Vitro Study

**DOI:** 10.3390/ijms24021401

**Published:** 2023-01-11

**Authors:** Jesús-David Tristán-López, Nereyda Niño-Martínez, Eleazar-Samuel Kolosovas-Machuca, Nuria Patiño-Marín, Idania De Alba-Montero, Horacio Bach, Gabriel-Alejandro Martínez-Castañón

**Affiliations:** 1 Doctorado Institucional en Ingeniería y Ciencia de Materiales, Universidad Autonoma de San Luis Potosi, Sierra Leona No. 550 Col. Lomas 2da. Sección, C. P., San Luis Potosí 78210, Mexico; 2 Facultad de Ciencias, Universidad Autonoma de San Luis Potosi, Av. Parque Chapultepec 1570, Privadas del Pedregal, C. P., San Luis Potosí 78295, Mexico; 3 Facultad de Estomatología, Universidad Autonoma de San Luis Potosi, Av. Dr. Manuel Nava No. 2, Zona Universitaria, C. P., San Luis Potosí 78290, Mexico; 4 Faculty of Medicine, Division of Infectious Diseases, University of British Columbia, Vancouver, BC V6H3Z6, Canada

**Keywords:** silver nanoparticles, orthodontic adhesives, adhesives, shear bond strength, bactericidal activity

## Abstract

There is a significant change in the bacterial plaque populations in the oral cavity during and after orthodontic treatment. Numerous studies have demonstrated that 2–96% of patients could increase the risk of white spot lesions. *Streptococcus mutans* and *Lactobacilli* ssp. are responsible for these white spot lesions. In this work, silver nanoparticles (AgNPs) with a diameter of 11 nm and dispersed in water were impregnated onto three different commercial orthodontic adhesives at 535 μg/mL. The shear bond strength (SBS) was assessed on 180 human premolars and metallic brackets. The premolars were divided into six groups (three groups for the commercial adhesives and three groups for the adhesives with AgNPs). All the groups were tested for their bactericidal properties, and their MIC, MBC, and agar template diffusion assays were measured. After adding AgNPs, the SBS was not significantly modified for any adhesive (*p* > 0.05), and the forces measured during the SBS did not exceed the threshold of 6 to 8 MPa for clinical acceptability in all groups. An increase in the bactericidal properties against both *S. mutans* and *L. acidophilus* was measured when the adhesives were supplemented with AgNPs. It was concluded that AgNPs can be supplement commercial orthodontic adhesives without modifying their mechanical properties with improved bactericidal activity.

## 1. Introduction

The oral cavity provides a suitable environment for the rapid growth of bacteria that demineralize the tooth enamel surface [1]. This demineralization is a common issue during fixed orthodontic treatment and is associated with developing dental plaques around the bracket [2]. Enamel demineralization is caused by organic acids produced by bacteria colonizing the brackets’ adjacent areas and are known as white spot lesions (WSL). These lesions are common problems in orthodontic procedures, and studies have reported a prevalence of 25% in a patient cohort of 885 patients [3]. Another study reported a prevalence of 38% and 46% in groups followed for six months (n = 37) and 12 months (n = 35), respectively [4]. Other studies have demonstrated that 2% to 96% of patients could increase the risk of WSL during orthodontic treatment [5].

The white and pale appearance of the enamel is due to the porosity that changes the enamel characteristics [6]. The main bacterial strains responsible for WSL are *Streptococcus mutans* and *Lactobacillus* ssp. [7].

WSL prevention is important to be performed at the beginning of the treatment, and some clinicians use a topical application to remineralize the surface as well as to promote natural remineralization using resin materials [8]. Although these are methods used to prevent or reduce the development of WSL, the cooperation of the patients in oral hygiene to prevent the development of oral biofilms through oral hygiene care is critical [9]. In recent years, researchers have centered on methods that do not require patient cooperation, such as adding antimicrobial agents to dental products, e.g., orthodontic adhesives [10]. Most of these attempts failed to achieve the goal due to the fast degradation of the products, releasing the antibacterial agents, causing low capability and safety concerns [11].

Nanotechnology has been applied to dental materials to increase the mechanical properties and anti-caries potential. Nanomaterials have great potential to reduce biofilm accumulation and restrain the demineralization process [12]. One of the most common nanomaterials used as antimicrobial agents are nanoparticles (NPs), which may provide a new strategy for the WSL treatment [11].

Metallic NPs, such as silver (Ag), zinc oxide (ZnO), and titanium (TiO_2_) have been used in the health arena due to their antimicrobial properties [13]. In dentistry, silver nanoparticles (AgNPs) have improved treatments with better outcomes, as they can be incorporated into adhesive materials in orthodontic treatment and other dentistry materials [14]. Recently, dental nanofiller and hydrophilic resin composites were introduced into the market because they have better stability, handling, and polishable properties. Moreover, nanofillers can decrease the surface roughness (SR) of orthodontic adhesives, one of the most significant factors in the bacterial adhesion and development of biofilms [15]. Very few studies have been performed to evaluate the bond strength and antimicrobial properties of nanofillers and hydrophilic orthodontic primers.

In the current study, the orthodontic bonding material strength and antimicrobial properties were assessed and compared by incorporating AgNPs into three different commercial hydrophilic and hydrophobic adhesive systems.

This study’s null hypothesis was that adding AgNPs would increase the antibacterial activity and would not make any differences in SBS between adhesives.

## 2. Results

### 2.1. AgNPs Characterization

**Figure 1:**
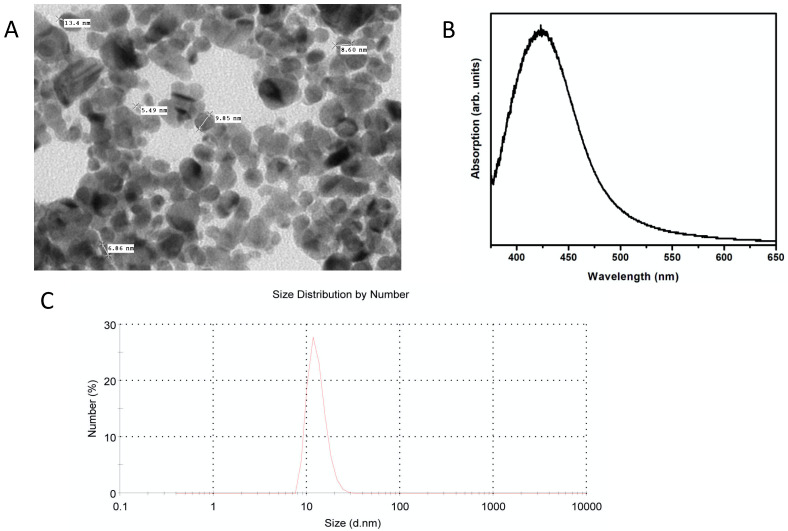
Characterization of the synthesized AgNPs. Synthesized AgNPs were characterized using (**A**) TEM imaging, (**B**) surface plasmon resonance, and (**C**) dynamic light scattering (DLS) for the determination of the hydrodynamic diameter.

Analysis of the AgNPs showed a spherical shape and a hydrodynamic diameter of 11 nm with a narrow particle size distribution (Figure 1A,B). The plasmon of the synthesized AgNPs was found at 415 nm (Figure 1C).

### 2.2. Shear Bond Strength (SBS) and Adhesive Remnant Index

**Figure 2:**
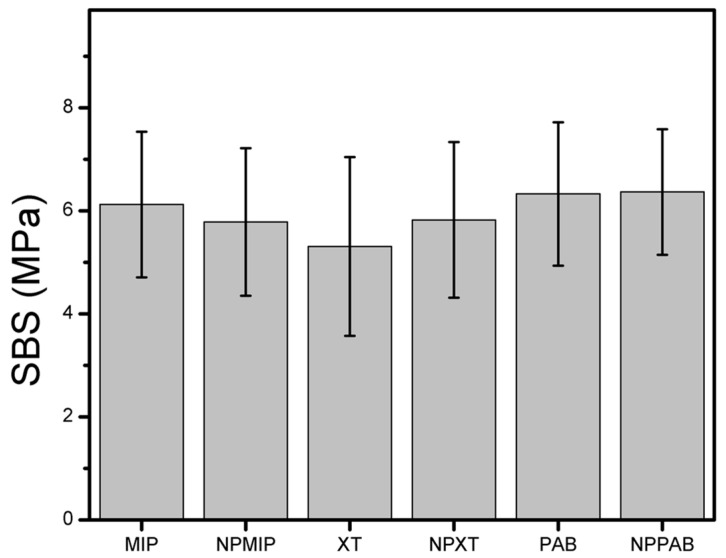
The mean SBS of the bracket to enamel in the six groups with a 95% confidence interval.

The results of the SBS are presented in Table 1 and Figure 2. The mean differences were analyzed with a two-tailed Student’s *t*-test using six degrees of freedom, assuming a normal distribution. Additionally, a Shapiro–Wilk test was used to determine the normal distribution. A statistical significance was considered when a *p* value of <0.05 was calculated. The SBS test showed no significant difference between the commercial adhesives with or without AgNPs.

**Figure 3:**
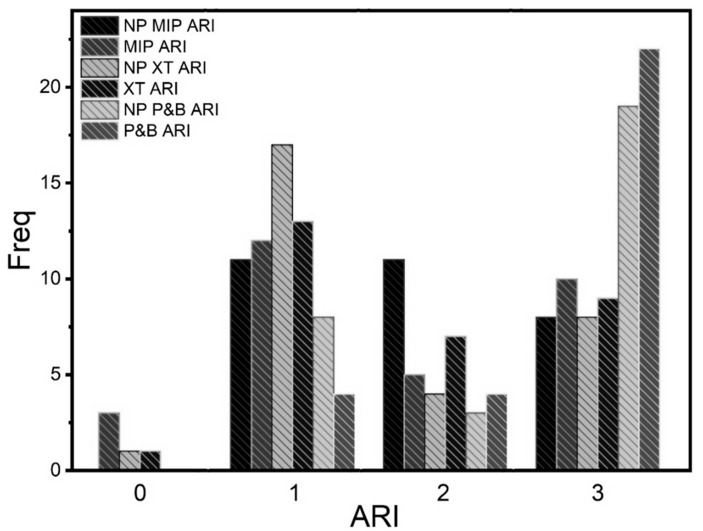
The frequency of adhesive remnant index (ARI) score number.

The frequency of adhesive failure in the six groups showed that mixed failure was most prevalent among the six groups (Figure 3 and Table 2).

### 2.3. Minimum Inhibitory Concentration and Minimal Bactericidal Concentration Determined by Broth Microdilution Assay

Analysis of the MIC and MBC results showed that MIC values decreased after adding AgNPs to the adhesives (*p* < 0.05) when tested against *L. acidophilus* (Table 3). When tested against *S. mutans*, the MIC values were higher (*p* < 0.05) or did not change (*p* > 0.05).

MBC values (Table 3) were obtained for the samples tested against *L. acidophilus* and *S. mutans*. For *L. acidophilus*, adding AgNPs into the adhesives improved bacterial growth inhibition in all of the samples (*p* < 0.05), but for *S. mutans* only the MIP with AgNPs added improved the antibacterial activity.

### 2.4. Agar Template Diffusion Assay

The antibacterial activity of the samples was tested by the diffusion assay to mimic the release of the antibacterial agent from the adhesives. The addition of AgNPs reduced bacterial growth, evidenced by larger inhibition halos compared to the adhesives without AgNPs. When AgNPs (535 μg/mL) were mixed in the adhesives, the inhibition halos ranged from 10.33 mm to 18.33 mm. In summary, all groups containing AgNPs presented a greater bacterial growth reduction than the adhesive groups without AgNPs (Table 4).

## 3. Discussion

Today there are new adhesives with hydrophilic technology and moisture properties, allowing the direct bonding procedure of the brackets to be relatively more straightforward. Another desirable property of orthodontic adhesives is to have antimicrobial properties since the brackets that are adhered to the enamel of the teeth remain in the mouth for at least two years. Due to this, it is common to find early carious lesions or WSL at the end of the orthodontic treatment [3].

In this study, extracted first and second premolars were stored in distilled water to avoid the alteration of the adhesion force [16]. However, other substances, such as artificial saliva, plain water, and saline solution, can be used for the same purpose [17].

It has been reported that enamel pretreatment can alter the results of the SBS test [18,19]; thus, unlike the current adhesion protocols that proposed 60 s for enamel etching [20], a traditional protocol with orthophosphoric acid and for only 15 s was used [21]. The rationale for this protocol is that it has been considered that a more extended period of etching or the use of different chemicals could be detrimental to the enamel properties. The forces measured in this study during the SBS did not exceed the threshold of 6 to 8 MPa for clinical acceptability in all groups, suggesting that the experimental primers (e.g., commercial adhesives with AgNPs added) can be used without any effect on the enamel [17,22].

Another study found a difference in the adhesion strength between conventional adhesives and adhesives supplemented with AgNPs contrasting the results of this study even though the preparation of the samples was similar [23], including the use of a similar universal machine. The notable difference could be due to the surface chemistry of the NPs. Whereas in the present study, AgNPs were surrounded by gallic acid molecules that do not interact with the molecules in the adhesives, the surface chemistry of the NPs used by Riad et al. [23] included polyvinyl pyrrolidone, which could interact with the chemistry of the adhesives [24]. Another study differed from the results reported here regarding a failure of the adhesive with AgNPs supplementation [25]. In the results reported here, differences were not found in SBS, and the characteristics of the primers can explain the differences in the results. For example, this study used several hydrophilic primers, whereas the other study used a hydrophobic primer [25].

Other researchers found a significant difference in the adhesive strength of adhesives with AgNPs in a higher concentration (713.3 μg/mL). In contrast, this study, a concentration of 535 μg/mL AgNPs was used for the three adhesives, with no alteration in the adhesive strength [26]. Similar results were obtained in the adhesive strength of adhesives supplemented with TiO_2_NPs [27]. However, the supplement of CuNPs, showed higher ARI scores, indicating a greater bonding force between the enamel and the adhesive. Although the force did not decrease adherence in our groups, the ARI was highly variable for all study groups [2].

Similar work observed significant differences when orthodontic primers were used in humid or dry conditions. Results showed a vast difference between both groups, decreasing the adhesive strength in the group with wet conditions. These results suggest that humidity control is necessary to avoid variations between the study groups [28]. Humidity control was considered in our study, which can explain the lack of significant differences in the adhesion tests.

Some concerns about the biocompatibility of AgNPs in orthodontics have been raised by researchers [29,30]. Previous studies from the authors of the present study evaluated the biocompatibility of these AgNPs [31,32], and reported cell viability percentages >95% when the AgNPs were tested against human dermal fibroblasts. Moreover, it was also reported that AgNP concentrations <4 μg/mL and at 0.5 μg/mL showed a cytotoxic effect that resulted in a death rate of 13.8% and <25%, respectively [33]. Moreover, in an in vivo study, the suspension of AgNPs did not generate any alterations in the outcome of the clinical characteristics, clinical chemistry, or hematology when Wistar rats were treated with AgNPs [34]. For these reasons, although the current study does not report biocompatibility tests, AgNPs are presented as a suitable option for orthodontic adhesion.

Lastly, when the same adhesives were not supplemented with AgNPs but used in a contaminated environment under humid and dry conditions, MIP and Transbond XT had a high adhesive strength even in a humid environment. These results are relevant because, in the present study, strict control of humidity was performed, supporting the inference that the adhesive strength was not affected in the study groups [17].

When mixing AgNPs with Transbond XT, Bahador et al. (2020) reported similar results to those reported here, the antimicrobial activity increased with a concentration-dependent effect [35]. On the other hand, Argueta-Figueroa et al. (2019) tested different concentrations of AgNPs with Transbond MIP and found that the adhesive did not present any antibacterial activity without AgNPs [36]. The differences with our study could be that the researchers did not use *S. mutans* or *L. acidophilus* but confirmed our results that the antibacterial activity of MIP was shown only when mixed with AgNPs. A similar study found similar results to the study presented here, but the authors used 50 nm AgNPs, whereas in the current study, 11 nm AgNPs were used with superior antibacterial activity [37].

The only commercial adhesive that showed antimicrobial activity for the antimicrobial test using the diffusion assay was the MIP, this can be explained by the presence of ethyl alcohol in the adhesive. However, ethyl alcohol does not have the same substantivity as AgNPs since the alcohol volatilizes immediately, and the AgNPs remain on the surface.

Further research is needed to remediate the limitations of this study, (i) inducing artificial demineralization around brackets, and (ii) introducing different concentrations of AgNPs to the adhesives.

## 4. Materials and Methods

### 4.1. Ethical Approval

The ethics committee of the Autonomous University of San Luis Potosi approved this study under protocol number CEI-FE-036-022.

### 4.2. Synthesis of AgNPs

AgNPs were prepared using an aqueous method after dissolving 0.169 g of AgNO_3_ in 90 mL of deionized water, followed by adding 0.1 g of gallic acid (previously dissolved in 10 mL of deionized water) under magnetic stirring. Finally, the pH value of the solution was raised to 10 using NaOH 3.0 M. After 20 min of continuous stirring; the AgNPs were ready for the characterization process. AgNPs were characterized using Vis–NIR spectroscopy, dynamic light scattering (DLS), and transmission electron microscopy (TEM).

A series of six adhesive samples were tested in the present study. The details of the materials are shown in Table 5.

### 4.3. Preparation of the Samples

One hundred eighty human premolars were obtained from patient extractions and stored in a standard saline solution (0.9% *w*/*v* of NaCl) with an osmolarity of 308 mOsm/L. The premolars included in this study did not show enamel defects or any previous restoration, including endodontic, orthodontic, or prosthetic treatment. The premolars were mounted individually in a self-curing acrylic block. Each premolar’s buccal crown surface was polished with fluoride-free pumice slurry and baking soda. After 180 stainless steel metal premolar brackets with a mesh base (TD Orthodontics, Monterrey, NL, Mexico) were straight bonded to the etched enamel. Three orthodontic primers with and without AgNPs (Transbond MIP, 3M^®^, Monrovia, CA, USA; Transbond XT, 3M^®^, Monrovia, CA, USA; Prime and Bond, Dentsply^®^, York Haven, PA, USA) and an orthodontic bonding system (Transbond XT, 3M^®^, Monrovia, CA, USA) were examined (six experimental groups; n = 30). Then, the enamel surface of each premolar was etched with 37% H_3_PO_4_ gel (Etching Acid, 3M, Monrovia, CA, USA) for 30 s, rinsed for 15 s, and dried with an air dental syringe for 20 s until the enamel had an opaque appearance. Each specimen group (n = 30) was bonded with the three primers without AgNPs, and the study groups (n = 30) were bonded with the same adhesives with added AgNPs. Transbond XT and 3M^®^ composite were spread onto the bracket base. After expelling the excess glue, the bracket was positioned on the tooth in the clinical crown center. After this, the bonding resin was removed using a dental explorer tool. Then, the composite was light-cured for 20 s from the occlusal surface. The bonding adhesives were all light-cured with a curing light (S10 Elipar; 3M/Unitek Dental Products), with a light intensity of 1000 mW/cm^2^ [38].

### 4.4. SBS

The samples were placed in a holder attached to the base plate of a universal testing machine (Mecmesin Omnitest Universal Machine, Slinfold, UK). A straight-edge plunger was mounted in the movable crosshead of the testing machine and positioned so that the leading edge was aimed at the enamel–composite interface before being brought into contact. A crosshead speed of 1 mm/min^2^ was performed, and the measurements were taken in Newton metrical units after the bracket was displaced. The bond strength was calculated in mPa using the formula:

Bond strength (mPa) = Force in Newtons/surface area of brackets in mm^2^

The surface area of the bracket was 9.75 mm^2^, as given by the manufacturer [39].

### 4.5. Adhesive Remnant Index

Each specimen was analyzed under a stereoscopic zoom microscope (SMZ800, Leica, Wetzlar, Germany) to identify the type of bond failure. Each sample was scored according to the amount of material remaining on the enamel surface as follows: (i) adhesive failure = no adhesive remaining, (ii) cohesive failure = all adhesive remaining, and (iii) mixed failure = only some of the adhesive remaining. Additionally, the residual composite remaining on the premolar was assessed by using the remnant index (ARI), where each specimen was scored according to the amount of material remaining on the enamel surface as follows: 0 = no adhesive remaining, 1 = less than 50% of the adhesive remaining, 2 = more than 50% of the adhesive remaining, and 3 = all adhesive remaining with a clear impression of the bracket base.

### 4.6. Determination of the Minimal Inhibitory and Bactericidal Concentrations

**Figure 4:**
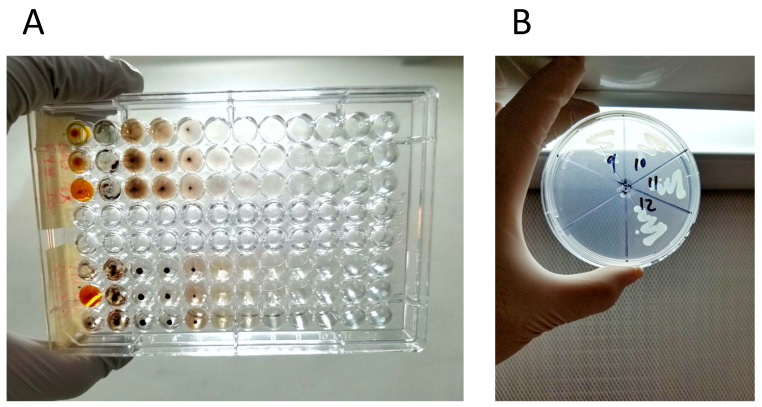
Determination of the (**A**) MIC and (**B**) MBC.

The antimicrobial activity of each sample was tested using the standard microdilution method, which determines the minimum inhibitory concentration (MIC) and the minimum bactericidal concentration (MBC), leading to the inhibition of bacterial growth [40]. The strains tested in this study were *L. acidophilus* (ATCC 4356) and *S. mutans* (ATCC 25175), using the McFarland scale. The bacterial concentration was standardized to an optical density between 0.2 and 0.215 at 568 nm (approximately 1 × 10^8^ CFU/mL). The samples in dispersion were diluted with 50 μL of Mueller–Hinton broth and 50 μL of phosphate buffer inoculated with the tested strains at a concentration of 1 × 10^5^ CFU/mL. Each plate was set up with AgNPs (1070 μg/mL) and broth as a positive and negative control, respectively. The plates were incubated at 35 ± 1 °C for 24 h. The MIC values were determined by visual examination, and the absence of turbidity or a “button” of growth at the bottom of the well was defined as growth inhibition (Figure 4A). The MBC value was determined, corresponding to the well with the minimum AgNPs concentration that prevented bacterial growth (Figure 4B).

### 4.7. Agar Template Diffusion Assay

The test was performed according to the methodology suggested by CCLS [40]. The same strains in the previous sections were used, and the bacterial suspension was adjusted to the 0.5 McFarland scale. Sterile swabs were used to seed bacteria onto the Petri dishes. The experimental groups contained the exact proportions of adhesives and AgNPs used in the mechanical test. The 9 mm discs were impregnated with 10 µL of the bonds to be tested (n = 3). The plates were incubated at 37 °C for 24 h. The growth diameter inhibition zones were horizontally and vertically measured with a vernier (Mitutoyo Corp., Kawasaki, Japan).

Biocompatibility tests for these AgNPs (spherical, 11 nm, and gallic acid reduced) are not reported in this work because they have been reported for our group in previous publications.

### 4.8. Statistical Analysis

Statistical analyses were performed with SPSS 13.0 for Windows (SPSS Inc. Chicago, IL, USA). For all variables, the normality and homogeneity were tested using the Shapiro–Wilk analysis. The results of the SBS were analyzed with a paired t-student test, using a significance level of 0.05. ARI is reported with descriptive statistics and frequency tables. MIC, MBC, and inhibition halos were analyzed using a Mann–Whitney U test.

## 5. Conclusions

It was concluded that hydrophilic and non-hydrophilic orthodontic adhesives with added AgNPs retain their mechanical properties and improve their antimicrobial activity against strains of interest in forming WSLs.

## Figures and Tables

**Table 1:** SBS (MPa) of the bracket to enamel in the groups (n = 30/group).

Group	Mean	SD	Minimum	Median	Maximum	*p* Value
MIP	6.12	1.41	3.75	5.57	8.73	0.364
NPMIP	5.78	1.43	2.04	5.65	9.27	
XT	5.31	1.73	2.34	5.05	8.72	0.225
NPXT	5.82	1.51	3.55	5.66	8.65	
PAB	6.32	1.39	3.35	6.53	8.96	0.913
NPPAB	6.36	1.21	3.52	6.60	9.16	

**Table 2:** The frequency of adhesive failure (AF) scores, number, and percentage in the groups (n = 30/group).

Group ARI Score	Adhesive	Mixed	Cohesive
MIP	3 (10%)	17 (57%)	10 (33%)
NPMIP	0 (0%)	22 (73%)	8 (27%)
XT	1 (3%)	20 (67%)	9 (30%)
NPXT	1 (3%)	21 (70%)	8 (27%)
PAB	0 (0%)	8 (27%)	22 (73%)
NPPAB	0 (0%)	11 (37%)	19 (63%)

**Table 3:** Minimum inhibitory concentration and minimal bactericidal concentration of the samples tested in this study.

			MIC (µg/mL)			MBC (µg/mL)		
Sample	*S. mutans*	*p*	*L. acidophilus*		*S. mutans*		*L. acidophilus*	*p*
MIP	66.87 ± 0.00	0.025	33.43 ± 0.00	0.025	16.17 ± 0.00	0.025	16.17 ± 0.00	0.025
NPMIP	133.75 ± 0.00		16.17 ± 0.00		2.02 ± 0.00		2.02 ± 0.00	
XT	16.17 ± 0.00	0.025	267.5 ± 0.00	0.025	8.08 ± 0.00	0.913	66.87 ± 0.00	0.025
NPXT	33.43 ± 0.00		4.04 ± 0.00		16.17 ± 0.00		0.50 ± 0.00	
PAB	33.43 ± 0.00	0.500	33.43 ± 0.00	0.025	2.02 ± 0.00	0.500	16.17 ± 0.00	0.025
NPPAB	33.43 ± 0.00		4.04 ± 0.00		2.02 ± 0.00		4.04 ± 0.00	
AgNPs (535 μg/mL) *	1.15 ± 0.00		0.39 ± 0.00		1.15 ± 0.00		0.39 ± 0.00	

MIC, minimum inhibitory concentration; MBC, minimum bactericide concentration. * Control.

**Table 4:** Agar template diffusion assay.

Sample	Halo Inhibition (mm)			
	*S. mutans*	*p*	*L. acidophilus*	*p*
MIP	10.33 ± 1.52	0.050	NA	0.034
NPMIP	18.33 ± 0.57		10.33 ± 0.33	
XT	NA	0.037	NA	0.025
NPXT	11 ± 1.0		9 ± 0.0	
PAB	NA	0.034	NA	0.025
NPPAB	10.33 ± 1.15		11 ± 0.0	

NA, no antibacterial activity observed.

**Table 5:** Composition of the adhesives used in this study.

Label	Name	Manufacturer	Composition
MIP	Transbond™ MIP	3M, Unitek, US	Ethyl alcohol, bisphenol A diglycidyl ether dimethacrylate, 2-hydroxyethyl methacrylate, 2-hydroxy-1,3-dimethacryloxypropane, copolymer of itaconic and acrylic acid, diurethane dimethacrylate, water, diphenyliodonium hexafluorophosphate, N,N-dimethylbenzocaine, triphenylantimony
NPMIP	Transbond™ MIP + AgNPs	3M, Unitek, US	Ethyl alcohol, bisphenol A diglycidyl ether dimethacrylate, 2-hydroxyethyl methacrylate, 2-hydroxy-1,3-dimethacryloxypropane, copolymer of itaconic and acrylic acid, diurethane dimethacrylate, water, diphenyliodonium hexafluorophosphate, N,N-dimethylbenzocaine, triphenylantimony, AgNPs
XT	Transbond™ XT	3M, Unitek, US	Silane-treated quartz (70–80%), bisphenol A diglycildyl ether dimethacrylate (10–20%), bisphenol A bis (2-hydroxyethyl ether) dimetacrylate (5–10%), silane treated silica (<2%)
NPXT	Transbond™ XT + AgNPs	3M, Unitek, US	Silane-treated quartz (70–80%), bisphenol A diglycildyl ether dimethacrylate (10–20%), bisphenol A bis (2-hydroxyethyl ether) dimetacrylate (5–10%), silane treated silica (<2%), AgNPs
PAB	Prime & Bond	Denstply, Sirona, US	PENTA, UDMA + T-resin (cross-linking agent) + D-resin (small hydrophilic molecule), butylated hydroxitoluene, 4-ethyl dimethyl aminobenzoate, cetilamine hydrofluoride, acetone, silica nanofiller
NPPAB	Prime & Bond + AgNPs	Denstply, Sirona, US	PENTA, UDMA + T-resin (cross-linking agent) + D-resin (small hydrophilic molecule), butylated hydroxitoluene, 4-ethyl dimethyl aminobenzoate, cetilamine hydrofluoride, acetone, silica nanofiller, AgNPs

PENTA: dipentaerythritol penta-acrylate phosphate; UDMA: Urethane dimethacrylate.
